# Genetic proxies for clinical traits are associated with increased risk of severe COVID-19

**DOI:** 10.1038/s41598-025-86260-z

**Published:** 2025-01-15

**Authors:** N. J. M. Chaddock, S. S. R. Crossfield, M. Pujades-Rodriguez, M. M. Iles, A. W. Morgan

**Affiliations:** 1 University of Leeds (School of Medicine and Leeds Institute for Data Analytics), Leeds, UK; 2 Leeds Teaching Hospitals NHS Trust (NIHR Leeds Biomedical Research Centre and NIHR Leeds Medtech and In vitro Diagnostics Co-operative), Leeds, UK

**Keywords:** Genetic association study, Medical genetics

## Abstract

Routine use of genetic data in healthcare is much-discussed, yet little is known about its performance in epidemiological models including traditional risk factors. Using severe COVID-19 as an exemplar, we explore the integration of polygenic risk scores (PRS) into disease models alongside sociodemographic and clinical variables. PRS were optimized for 23 clinical variables and related traits previously-associated with severe COVID-19 in up to 450,449 UK Biobank participants, and tested in 9,560 individuals diagnosed in the pre-vaccination era. Associations were further adjusted for (i) sociodemographic and (ii) clinical variables. Pathway analyses of PRS were performed to improve biological understanding of disease. In univariate analyses, 17 PRS were associated with increased risk of severe COVID-19 and, of these, four remained associated with COVID-19 outcomes following adjustment for sociodemographic/clinical variables: hypertension PRS (OR = 1.1, 95%CI 1.03–1.18), atrial fibrillation PRS (OR = 1.12, 95%CI 1.03–1.22), peripheral vascular disease PRS (OR = 0.9, 95%CI 0.82–0.99), and Alzheimer’s disease PRS (OR = 1.14, 95%CI 1.05–1.25). Pathway analyses revealed enrichment of genetic variants in pathways for cardiac muscle contraction (genes *N* = 5; *beta[SE]* = 3.48[0.60]; adjusted*-P* = 1.86 × 10^−5^). These findings underscore the potential for integrating genetic data into epidemiological models and highlight the advantages of utilizing multiple trait PRS rather than a single PRS for a specific outcome of interest.

**Supplementary Information:**

The online version contains supplementary material available at 10.1038/s41598-025-86260-z.

## Introduction

As genetic testing becomes more cost-effective, interest has grown over its potential utility in epidemiological modelling and ultimately clinical care. Indeed, its use in healthcare has become increasingly common over the past decade, with polygenic risk scores (PRS) developed for several diseases and the first pilot study introducing PRS to clinical practice currently being performed in the UK National Health Service (NHS)^1–5^. However, limited evidence exists regarding the performance of genetic data in epidemiological models when incorporated alongside common sociodemographic and clinical variables.

There is great potential value in integrating genetic data into epidemiological studies, particularly for complex diseases that are influenced by heritable risk factors. An individual’s germline genotype data could be used to develop a proxy measure of their propensity to develop each of these risk traits. The use of PRS, which summarise an individual’s genetic propensity to a trait^6^, may reduce the need for time-consuming collection of clinical data and minimize the impact of human bias on disease risk modelling.

Clinical traits and biomarkers may not always be available in electronic health records, and can be difficult to collect consistently across institutions and countries, due to variation in diagnostic criteria and subjective clinical decision making^7,8^. Predictive models based on historical clinical diagnostic records may also miss a large section of the affected or at-risk population, including those with a high probability of developing future disease but lacking diagnoses (e.g. pre-diabetics^8^), limiting their efficacy. Therefore, the use of PRS as genetic proxies for both the disease of interest and for related traits where possible could benefit epidemiological studies and ultimately healthcare systems.

Coronavirus disease 2019 (COVID-19), the condition caused by the spread of the highly transmissible severe acute respiratory syndrome coronavirus 2 (SARS-CoV-2), had a devastating effect on health and economies worldwide^9^. However, the rapid scientific response to the COVID-19 pandemic has resulted in a wealth of genetic and clinical data, from which several sociodemographic (e.g. obesity, male sex, older age), clinical (e.g. diabetes, comorbidity count) and genetic risk factors for poor COVID-19 outcomes have been identified^2,10–19^. The large volume of data generated during the COVID-19 pandemic, and the strong genetic component observed in COVID-19 outcomes (with heritability estimates of up to 41% for COVID-19 severity)^20^, make it an ideal case study to investigate the integration of PRS into epidemiological models.

In this study, we tested several trait PRS for associations with hospitalization, critical care admission and death from COVID-19, and sequentially adjust associations for sociodemographic and clinical variables. We highlight the value of integrating genetic data into epidemiological models along with established risk factors, and use in silico pathway analyses of PRS to reveal a shared aetiology of traits which could be leveraged to provide better insights into disease pathogenesis.

## Methods

### Data source

This study was approved by UK Biobank (Application 24559), the population-based cohort that links sources of biological and phenotypic data on > 500,000 individuals in the UK. All methods were performed in accordance with the relevant guidelines and regulations. Self-report questionnaires and baseline biological measurements were recorded from the years 2006–2010, when participants (then aged 40–69 years) were recruited^21^.

### Study population

**Fig. 1:**
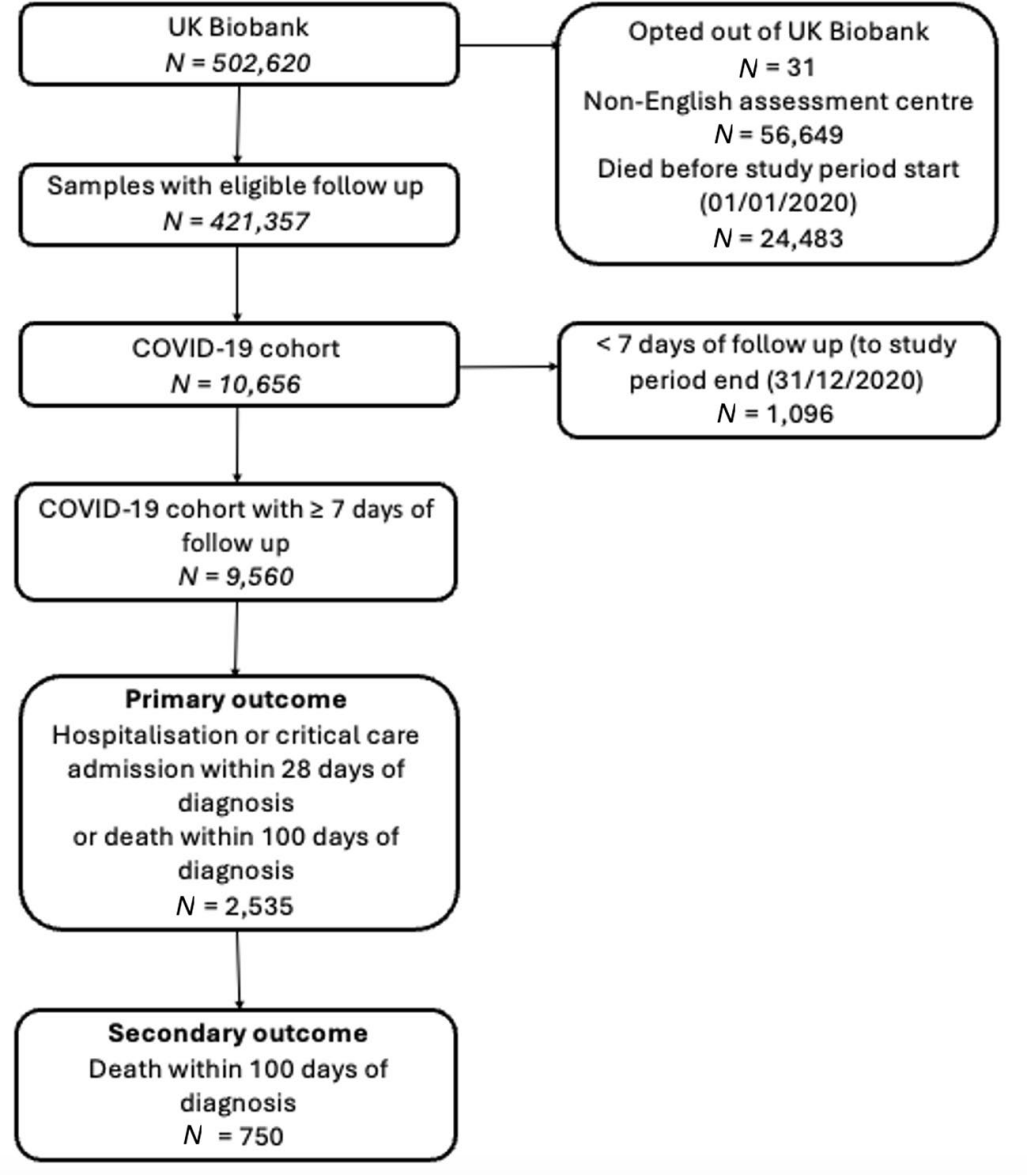
Summary of the cohort selection flow used in this study.

Details of the study population and COVID-19 datasets used in this work may be found in Crossfield et al. (2022)^2^. To summarize, UK Biobank participants with baseline assessment data, who passed genetic quality control (QC) were included in the study. Individuals included were from assessment centres in England, alive at the start of the study period (1 January 2020) and had not withdrawn consent (Fig. 1). COVID-19 diagnosis was defined as ICD-10 code U071 or U072 from hospital or death certificate data, or a positive laboratory test result. Furthermore, both a transethnic population and a “white European” subpopulation were included in the study. The white European subpopulation was defined as those who lay within the European genetic principal component (PC) cluster, as well as having one of several self-reported “white European” ethnicities in baseline data (*n* = 404,534/450,577 = 89.78% of entire cohort)^2^.

### Study outcomes

The primary outcome for this study was severe COVID-19, a composite formed from those with a hospital or critical care admission within 28 days of COVID-19 diagnosis (including admissions 1–3 days preceding diagnosis, to account for laboratory testing delays), with a secondary outcome of death within 100 days of COVID-19 diagnosis. Disease controls were defined as those who had a COVID-19 diagnosis but were not hospitalized, had no critical care admission (both within 28 days), and did not die within 100 days following diagnosis. All analyses were performed in both a transethnic cohort (2,109 cases and 5,970 controls for severe COVID-19 and 636 cases and 7,443 controls for COVID-19 mortality) and the white European subset of these (1,833 cases and 5,162 controls for severe COVID-19 and 570 cases and 6,425 controls for COVID-19 mortality).

### Variable selection

Clinical variables, including related traits, were selected for PRS modelling based on our previous COVID-19 severity and mortality models^2^. Additional covariates included previously defined sociodemographic variables (e.g. age and Townsend deprivation index), a previously developed COVID-19 PRS optimised in a white European population (hereafter named “PRS_e2_” maintaining the nomenclature used in our original publication) and selected clinical variables and related traits based on prior observational evidence (e.g. cardiovascular disease [CVD], angina, and comorbidity count; Supplementary Methods; Supplementary Tables 1–2)^2^.

### Statistical analyses

Statistical analyses were performed in R v3.6.2^22^ to model the risk of severe COVID-19 using logistic regression, and model risk of death (over a period of 100 days post-diagnosis) using Cox proportional hazards regression. Details regarding the modelling of specific variables may be found in Supplementary Methods.

### Polygenic risk score associations

**Fig. 2:**
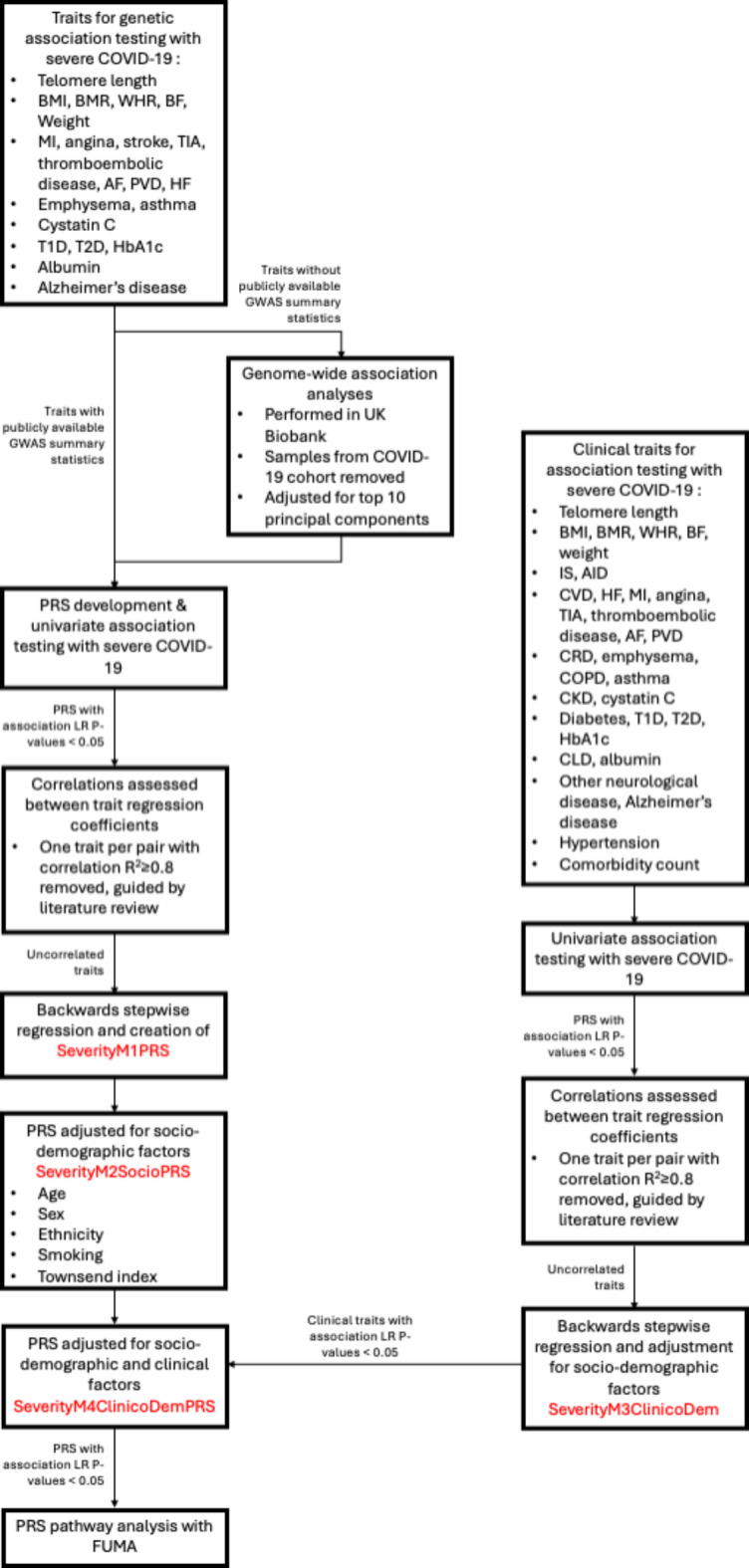
Outline of the analysis steps taken in this study. BMI, body mass index; BMR, basal metabolic rate; WHR, waist-hip ratio; BF, body fat percentage; MI, myocardial infarction; TIA, transient ischaemic attacks; AF, atrial fibrillation; PVD, peripheral vascular disease; HF, heart failure; T1D, type 1 diabetes; T2D, type 2 diabetes; HbA1c, glycated haemoglobin; GWAS, genome-wide association study; FUMA, Functional Mapping and Annotation of Genome-Wide Association Studies; CVD, cardiovascular disease; CRD, chronic respiratory disease; COPD, chronic obstructive pulmonary disease; CKD, chronic kidney disease; CLD, chronic liver disease.

PRS were optimized for prediction of the selected clinical variables and related traits in an independent cohort and then tested for association with severe COVID-19 **(**Fig. 2**)**. Details of QC and PRS optimization are outlined in Supplementary Methods. Briefly, for each PRS, a genome-wide association study (GWAS) was performed in PLINK v1.9^23^, regressing the phenotype on each genetic variant using either linear or logistic regression in the white European subpopulation, including the top 10 PCs from principal component analysis (PCA) as covariates to adjust for population stratification. Samples from the COVID-19 cohort were removed from the UK Biobank cohort prior to trait GWAS analyses, to ensure no overlap between the cohorts at the PRS optimization stage. Summary statistics from each clinical variable GWAS were provided as training datasets to optimize PRS using the clumping and thresholding approach implemented in PRSice v2.3.3, adjusting for the top 10 PCs from PCA as covariates^24^.

PRS were then tested for association with each COVID-19 outcome in univariate analyses (in both the transethnic and white European cohorts) and those PRS with a likelihood ratio (LR) test *P-value* < 0.05 were combined in a model of severe COVID-19, and another of COVID-19 mortality. To remove highly correlated PRS, a correlation matrix was formed using the regression coefficients from each of these models separately, and one PRS from each pair with a regression coefficient correlation *R*^2^ ≥ 0.8 was removed, retaining the most clinically relevant trait guided by a review of the literature. To further refine the model and remove redundant variables, backwards stepwise regression was performed and PRS with a LR test *P* < 0.05 were retained in the models (henceforth known as “SeverityM1PRS” and “MortalityM1PRS”; Supplementary Methods). PRS odds ratios (ORs) are reported per unit change in standard deviation.

### Adjustment for sociodemographic and clinical covariates

PRS in the SeverityM1PRS and MortalityM1PRS models were then adjusted for previously reported socio-demographic variables^2^, creating “SeverityM2SocioPRS” and “MortalityM2SocioPRS” respectively.

Prior to adjustment of PRS for clinical variables and related traits in our models, univariate analyses were performed to identify clinical traits associated with COVID-19 severity or COVID-19 mortality, and variables with a LR test *P-value* of < 0.05 were further adjusted for sociodemographic variables. To remove highly correlated clinical variables, a correlation matrix was formed using the regression coefficients of the clinical variables and one variable from each pair with a regression coefficient correlation *R*^2^ ≥ 0.8 was removed, guided by a review of the literature. Removal of redundant variables was then performed using backwards stepwise regression, with sociodemographic variables retained in models due to prior evidence of COVID-19 outcome associations, creating “SeverityM3ClinicoDem” and “MortalityM3ClinicoDem” (Supplementary Methods).

### Clinico-demographic adjusted PRS associations

Finally, PRS associations in SeverityM2SocioPRS were further adjusted for clinical factors which had a LR test *P-value* < 0.05 in earlier analyses, creating the “SeverityM4ClinicoDemPRS” model. This was repeated for PRS associated with COVID-19 mortality in MortalityM2SocioPRS, creating “MortalityM4ClinicoDemPRS” (Supplementary Methods).

### Comparisons of model fit

To compare the epidemiological models created in this work, model fit was assessed using the Akaike Information Criterion (AIC) and Bayesian Information Criterion (BIC) statistics. This was repeated to compare models for the COVID-19 mortality outcome.

### Pathway analysis

Pathway analysis was performed on those PRS associated with COVID-19 outcomes in the final SeverityM4ClinicoDemPRS and MortalityM4ClinicoDemPRS models. This was conducted using the Functional Mapping and Annotation of Genome-Wide Association Studies (FUMA) v1.4.0 tool^25^, a package that combines multiple in-silico tools (including the Multi-marker Analysis of GenoMic Annotation (MAGMA) gene-based test^26^) to provide functional interpretation of SNPs in PRS. SNPs analysed by FUMA were restricted to loci found in each PRS, and linkage disequilibrium (LD) thinning was performed using the same parameters as PRSice (*R*^2^ < 0.1 in 250 kb blocks) and the 1000 Genomes Phase 3 European panel as reference. More information may be found in the Supplementary Methods.

## Results

### Polygenic risk score associations

GWAS were performed for 23 UK Biobank clinical variables and related traits, identifying a total of 41,530 independent (LD *R*^2^ < 0.6) SNP associations (*P* < 5 × 10^− 8^) (Supplementary Table 1). PRS were then optimized using summary statistics produced by these analyses, adjusting for 10 PCs, and associations were found between 17 PRS and COVID-19 outcomes in univariate analyses (Table 1; Supplementary Tables 3–16).

**Table 1:** Univariate PRS associations with COVID-19 severity and COVID-19 mortality in both the transethnic and white European populations.

Variable	COVID-19 Severity				COVID-19 Mortality			
	Transethnic		White European		Transethnic		White European	
	OR(95%CI)	*P*(LR-test)	OR(95%CI)	*P*(LR-test)	OR(95%CI)	*P*(LR-test)	OR(95%CI)	*P*(LR-test)
Telomere length PRS	0.92 (0.87–0.97)	0.004	0.92 (0.87–0.98)	0.007	0.92 (0.84–1.01)	0.066	0.95 (0.86–1.04)	0.231
BMI PRS	1.17 (1.1–1.24)	< 0.001	1.18 (1.11–1.25)	< 0.001	1.08 (0.99–1.18)	0.079	1.08 (0.99–1.18)	0.099
BMR PRS	1.11 (1.05–1.17)	< 0.001	1.12 (1.05–1.18)	< 0.001	1.05 (0.96–1.14)	0.311	1.05 (0.96–1.15)	0.312
WHR PRS	1.11 (1.05–1.17)	< 0.001	1.12 (1.05–1.19)	< 0.001	1.04 (0.95–1.13)	0.415	1.04 (0.95–1.14)	0.417
Weight PRS	1.13 (1.07–1.19)	< 0.001	1.14 (1.08–1.21)	< 0.001	1.08 (0.99–1.17)	0.098	1.07 (0.98–1.17)	0.146
BF PRS	1.12 (1.06–1.18)	< 0.001	1.13 (1.06–1.19)	< 0.001	1.04 (0.95–1.13)	0.374	1.04 (0.95–1.14)	0.368
MI PRS	1.09 (1.03–1.15)	3 × 10^− 3^	1.09 (1.02–1.15)	0.007	1.03 (0.95–1.13)	0.479	1.03 (0.94–1.13)	0.500
Angina PRS	1.09 (1.03–1.15)	0.003	1.1 (1.04–1.17)	0.002	1.06 (0.98–1.16)	0.161	1.05 (0.96–1.16)	0.262
Stroke PRS	1.1 (1.04–1.16)	< 0.001	1.12 (1.06–1.19)	< 0.001	1.07 (0.98–1.17)	0.108	1.08 (0.98–1.18)	0.110
TIA PRS	1.04 (0.98–1.1)	0.166	1.02 (0.96–1.09)	0.447	0.98 (0.89–1.06)	0.572	0.97 (0.89–1.06)	0.535
Thromboembolic PRS	1.02 (0.97–1.08)	0.389	1.02 (0.96–1.08)	0.496	0.99 (0.91–1.09)	0.902	0.99 (0.9–1.08)	0.768
AF PRS	1.06 (1.01–1.12)	0.032	1.07 (1.01–1.14)	0.022	1.15 (1.06–1.26)	0.001	1.15 (1.05–1.26)	0.002
PVD PRS	0.96 (0.91–1.02)	0.153	0.96 (0.9–1.01)	0.136	0.93 (0.85–1.02)	0.102	0.91 (0.83–0.99)	0.037
HF PRS	1.04 (0.99–1.1)	0.126	1.05 (0.99–1.11)	0.108	1.01 (0.93–1.1)	0.782	1 (0.92–1.1)	0.950
Emphysema PRS	1.05 (0.99–1.11)	0.077	1.04 (0.98–1.11)	0.149	1.01 (0.92–1.1)	0.873	0.99 (0.9–1.08)	0.786
Asthma PRS	1 (0.95–1.07)	0.774	1.02 (0.96–1.08)	0.505	1.01 (0.93–1.1)	0.815	1 (0.91–1.1)	0.989
Cystatin C PRS	1.07 (1.01–1.13)	0.021	1.06 (1–1.13)	0.039	1.05 (0.96–1.15)	0.255	1.05 (0.95–1.15)	0.332
T1D PRS	1.04 (0.99–1.1)	0.133	1.04 (0.98–1.1)	0.187	1.04 (0.95–1.13)	0.408	1.01 (0.92–1.11)	0.852
T2D PRS	1.13 (1.07–1.19)	< 0.001	1.14 (1.07–1.21)	< 0.001	1.06 (0.97–1.16)	0.178	1.08 (0.98–1.18)	0.111
HbA1c PRS	1.1 (1.04–1.16)	< 0.001	1.11 (1.05–1.18)	< 0.001	1.03 (0.94–1.12)	0.561	1.04 (0.95–1.14)	0.376
Hypertension PRS	1.15 (1.08–1.21)	< 0.001	1.15 (1.08–1.22)	< 0.001	1.08 (0.99–1.18)	0.085	1.07 (0.98–1.18)	0.125
Albumin PRS	0.9971 (0.9432–1.0541)	0.919	0.9957 (0.9384–1.0566)	0.888	0.99 (0.91–1.08)	0.884	1 (0.91–1.09)	0.951
Alzheimer’s PRS	1.03 (0.97–1.09)	0.298	1.03 (0.97–1.09)	0.303	1.16 (1.07–1.26)	6.04 × 10^− 4^	1.16 (1.06–1.26)	0.002

Severe COVID-19: hospitalization or critical care admission within 28 days of COVID-19 diagnosis or death within 100 days of COVID-19 diagnosis.

COVID-19 mortality: death within 100 days of COVID-19 diagnosis.

*P*-value from the likelihood ratio test for association.

OR, odds ratio; HR, hazard ratio; CI, confidence intervals; *P*, *P*-value; LR, likelihood ratio; PRS, polygenic risk score; BMI, body mass index; BMR, basal metabolic rate; BF%, body fat percentage; WHR, waist-hip ratio; IS, immunosuppressant; CVD, cardiovascular disease; MI, myocardial infarction; HF, heart failure; TIA, transient ischaemic attack; AF, atrial fibrillation; PVD, peripheral vascular disease; HbA1c, glycated haemoglobin; T1D, type 1 diabetes; T2D, type 2 diabetes; COPD, chronic obstructive pulmonary disease; PRSe2, PRSe2, White European polygenic risk score 2.

### Adjustment for sociodemographic and clinical variables

We then sought to determine whether clinical trait PRS were associated with the COVID-19 outcomes and whether these associations persisted after adjustment for known sociodemographic variables.

No PRS were found to be highly correlated (*R*^2^ > 0.8). Following removal of redundant PRS using backwards stepwise regression (SeverityM1PRS), and adjustment for sociodemographic variables in the SeverityM2SocioPRS, three PRS remained associated with severe COVID-19 in the transethnic and/or white European models (Supplementary Table 17): BMI PRS (adjusted odds ratio [AOR] = 1.14 95% confidence intervals [CI] 1.07–1.21, *P-value* [*P*] = 9.51 × 10^− 5^ [transethnic]; AOR = 1.15, 95%CI 1.07–1.23, *P* = 8.00 × 10^− 5^ [white European]), stroke PRS (AOR = 1.08, 95%CI 1.01–1.15, *P* = 0.02 [white European]) and hypertension PRS (AOR = 1.11, 95%CI 1.04–1.18, *P* = 2.63 × 10^− 3^ [transethnic]; AOR = 1.09, 95%CI 1.02–1.17, *P* = 0.01 [white European]). More details of correlations, backwards stepwise regression and COVID-19 mortality models (MortalityM1PRS and MortalityM2SocioPRS) may be found in Supplementary Results.

Details of PRS associations with COVID-19 mortality in MortalityM2SocioPRS (Supplementary Table 18) may be found in Supplementary Results. Associated PRS included the AF PRS (AOR = 1.12, 95%CI 1.03–1.22, *P* = 0.01 [transethnic]; AOR = 1.11, 95%CI 1.02–1.22, *P* = 0.02 [white European]), the PVD PRS (AOR = 0.9, 95%CI 0.83–0.99, *P* = 0.03 [white European]), and the Alzheimer’s disease PRS (AOR = 1.14, 95%CI 1.05–1.24, *P* = 2.50 × 10^− 3^ [transethnic]; AOR = 1.14, 95%CI 1.04–1.25, *P* = 4.44 × 10^− 3^ [white European]). Of note, “PRS_e2_” was no longer significant in these models.

To select clinical variables/traits for further adjustment of our SeverityM2SocioPRS and MortalityM2SocioPRS models, univariate associations between severe COVID-19 and clinical variables were defined, sociodemographic factors were included in the models (SeverityM3ClinicoDem and MortalityM3ClinicoDem) and highly correlated and residual redundant clinical variables were sequentially removed (Supplementary Tables 19–21).

After the PRS associations were further adjusted for clinical variables in SeverityM4ClinicoDemPRS, one PRS remained associated with severe COVID-19 (Table 2): the hypertension PRS (AOR = 1.1, 95%CI 1.03–1.18, *P* = 4.83 × 10^− 3^ [transethnic]). An additional three PRS were associated with COVID-19 mortality in the MortalityM4ClinicoDemPRS, including the Alzheimer’s PRS (AOR = 1.14, 95%CI 1.05–1.25, *P* = 2.54 × 10^− 3^ [transethnic] and AOR = 1.14, 95%CI 1.04–1.25, *P* = 5.22 × 10^− 3^ [white European]), AF PRS (AOR = 1.12, 95%CI 1.03–1.22, *P* = 9.98 × 10^− 3^ [transethnic] AOR = 1.13, 95%CI 1.03–1.23, *P* = 0.11 [white European]) and PVD PRS in the white European population (AOR = 0.9, 95%CI 0.82–0.99, *P* = 0.02) (Table 3).

**Table 2:** Clinico-demographic and PRS adjusted odds ratios of severe COVID-19 associations in patients diagnosed with COVID-19 in the transethnic (*N* = 6,462) and white European (*N* = 5,632) cohorts. SeverityM5ClinicoPRS model, including variables with statistically significant severe COVID-19 associations in SeverityM3SocioPRS and SeverityM4Clinical.

Variable	Transethnic		White European	
	OR(95%CI)	*P*(LR-test)	OR(95%CI)	*P*(LR-test)
Age				
1	1	2.00 × 10^− 98^	1	7.26 × 10^− 80^
2	0.95 (0.72–1.25)		1.12 (0.83–1.5)	
3	1.8 (1.42–2.29)		2.02 (1.54–2.67)	
4	3.82 (3.02–4.87)		4.07 (3.1–5.37)	
5	6.04 (4.75–7.73)		6.01 (4.59–7.93)	
Telomere length				
1	1	3.28 × 10^− 3^	1	0.02
2	0.91 (0.75–1.09)		0.91 (0.75–1.12)	
3	1 (0.83–1.21)		1.07 (0.87–1.31)	
4	0.78 (0.63–0.95)		0.82 (0.66–1.01)	
5	0.82 (0.67–1)		0.84 (0.67–1.04)	
Sex				
Female	1	4.32 × 10^− 11^	1	3.53 × 10^− 4^
Male	1.53 (1.34–1.74)		1.54 (1.16–2.05)	
Ethnicity				
White	1	6.59 × 10^− 3^	NA	NA
Black	2.03 (1.32–3.1)			
Other	0.98 (0.73–1.3)			
Smoking				
Never	1	1.34 × 10^− 6^	1	1.21 × 10^− 5^
Former	1.11 (0.97–1.28)		1.06 (0.91–1.23)	
Current	1.62 (1.33–1.98)		1.63 (1.31–2.02)	
Townsend				
1	1	1.73 × 10^− 5^	1	1.39 × 10^− 3^
2	0.94 (0.76–1.17)		0.93 (0.74–1.16)	
3	0.78 (0.63–0.98)		0.79 (0.63–0.99)	
4	1.06 (0.86–1.3)		1.11 (0.9–1.39)	
5	1.31 (1.07–1.6)		1.21 (0.98–1.5)	
BMI				
1	1	2.26 × 10^− 7^	NA	NA
2	1.01 (0.81–1.26)			
3	1.06 (0.85–1.31)			
4	1.12 (0.9–1.39)			
5	1.6 (1.28–2)			
BMI PRS^^^	1.03 (0.96–1.1)	0.370	1.05 (0.97–1.12)	0.220
BF%				
	NA	NA	1	4.44 × 10^− 3^
			1.18 (0.94–1.48)	
			1.12 (0.88–1.43)	
			1.4 (1.06–1.87)	
			1.56 (1.12–2.16)	
WHR				
	NA	NA	1	0.040
			1.01 (0.78–1.3)	
			1.26 (0.97–1.65)	
			1.45 (1.09–1.94)	
			1.42 (1.04–1.95)	
IS	2.18 (1.57–3.04)	2.42 × 10^− 5^	2.09 (1.46–2.98)	9.44 × 10^− 5^
CVD	NA	NA	1.35 (1.12–1.63)	0.002
Stroke PRS^^^	NA	NA	1.05 (0.99–1.13)	0.120
Emphysema	2.06 (1.31–3.27)	1.71 × 10^− 3^	1.85 (1.15–3.02)	0.010
COPD	4.22 (1.84–11)	7.01 × 10^− 4^	4.38 (1.83–12.23)	1.10 × 10^− 3^
Cystatin C				
1	1	5.30 × 10^− 11^	1	9.05 × 10^− 9^
2	1.18 (0.93–1.5)		1.14 (0.88–1.48)	
3	1.46 (1.16–1.86)		1.43 (1.11–1.84)	
4	1.42 (1.12–1.81)		1.36 (1.05–1.75)	
5	2.19 (1.73–2.79)		2 (1.55–2.59)	
Diabetes	NA	NA	1.7 (1.28–2.26)	0.02
HbA1c				
1	1	4.41 × 10^− 10^	1	7.31 × 10^− 3^
2	1.1 (0.88–1.38)		1.04 (0.81–1.33)	
3	1.17 (0.94–1.46)		1.08 (0.86–1.37)	
4	1.34 (1.08–1.66)		1.3 (1.03–1.64)	
5	1.73 (1.4–2.16)		1.34 (1.06–1.71)	
Hypertension PRS^^^	1.1 (1.03–1.18)	4.83 × 10^− 3^	1.07 (1–1.15)	0.060
Other neurological	1.92 (1.3–2.84)	1.25 × 10^− 3^	1.91 (1.28–2.86)	2.30 × 10^− 3^
Comorbidity count*				
1	1	1.16 × 10^− 4^	1	3.79 × 10^− 5^
2	1.22 (1.05–1.41)		1.23 (1.06–1.42)	
3	1.50 (1.24–1.81)		1.53 (1.27–1.85)	

Severe COVID-19: hospitalization or critical care admission within 28 days of COVID-19 diagnosis or death within 100 days of COVID-19 diagnosis.

Clinico-demographic and PRS-adjusted model included age (as continuous), sex, ethnicity (in transethnic population), smoking status, Townsend deprivation quintile, immunosuppressant use, co-morbidities, anthropomorphic and biochemical traits (each as continuous), and PRS (as continuous). In this model, co-morbidity count is adjusted for these variables excepting the following comorbidities: cardiovascular disease, diabetes and neurological disease.

*Count of the following co-morbidities: cardiovascular disease, chronic respiratory disease, chronic kidney disease, diabetes, hypertension, chronic liver disease, neurological disease.

^^^Adjusted for top 10 principal components from principal component analysis.

*P*-value from the likelihood ratio test for association.

OR, odds ratio; CI, confidence intervals; *P*, *P*-value; LR, likelihood ratio; PRS, polygenic risk score; BMI, body mass index; BMR, basal metabolic rate; BF%, body fat percentage; WHR, waist-hip ratio; IS, immunosuppressant; CVD, cardiovascular disease; COPD, chronic obstructive pulmonary disease; HbA1c, glycated haemoglobin.

**Table 3:** Clinico-demographic and PRS adjusted hazard ratios of COVID-19 mortality associations in patients diagnosed with COVID-19 in the transethnic (*N* = 6,462) and white European (*N* = 5,632) cohorts. MortalityM5ClinicoPRS model, including variables with statistically significant COVID-19 mortality associations in MortalityM3SocioPRS and MortalityM4Clinical.

Variable	Transethnic		White European	
	HR(95%CI)	*P*(LR-test)	HR(95%CI)	*P*(LR-test)
Age				
1	1	< 2.2 × 10^− 16^	1	< 2.2 × 10^− 16^
2	2.18 (0.94–5.06)		1.57 (0.67–3.67)	
3	5.44 (2.59–11.44)		3.99 (1.88–8.48)	
4	11.3 (5.47–23.34)		8.3 (3.99–17.29)	
5	23.53 (11.44–48.4)		16.34 (7.91–33.79)	
Telomere length				
1	1	1.42 × 10^− 4^	1	4.56 × 10^− 4^
2	0.78 (0.62–0.99)		0.79 (0.61–1.02)	
3	0.82 (0.64–1.05)		0.86 (0.66–1.12)	
4	0.51 (0.38–0.7)		0.56 (0.41–0.77)	
5	0.68 (0.5–0.92)		0.68 (0.49–0.94)	
Sex				
Female	1	8.65 × 10^− 5^	1	2.75 × 10^− 4^
Male	1.48 (1.21–1.79)		1.6 (1.13–2.26)	
Ethnicity				
White	1	0.03	NA	NA
Black	1.75 (0.97–3.18)			
Other	0.81 (0.49–1.36)			
Smoking				
Never	1	5.17 × 10^− 6^	1	7.98 × 10^− 6^
Former	1.15 (0.94–1.41)		1.18 (0.95–1.46)	
Current	1.77 (1.36–2.31)		1.86 (1.41–2.46)	
Townsend				
1	1	1.76 × 10^− 2^	1	0.04
2	0.8 (0.59–1.1)		0.77 (0.56–1.07)	
3	0.8 (0.59–1.08)		0.86 (0.62–1.17)	
4	0.99 (0.74–1.32)		1.02 (0.75–1.38)	
5	0.99 (0.75–1.31)		0.99 (0.74–1.32)	
BMI				
1	1	7.64 × 10^− 2^	NA	NA
2	0.98 (0.72–1.34)			
3	0.75 (0.55–1.02)			
4	0.76 (0.56–1.04)			
5	1.09 (0.8–1.48)			
BMR				
1	NA	NA	1	5.53 × 10^− 3^
2			0.73 (0.51–1.05)	
3			0.86 (0.57–1.28)	
4			0.69 (0.44–1.08)	
5			0.57 (0.35–0.93)	
WHR				
1	NA	NA	1	0.02
2			0.89 (0.59–1.35)	
3			1.07 (0.7–1.65)	
4			1.17 (0.75–1.82)	
5			1.36 (0.86–2.15)	
AF PRS^^^	1.12 (1.03–1.22)	9.98 × 10^− 3^	1.13 (1.03–1.23)	0.01
PVD	3.28 (1.21–8.89)	0.03	2.99 (1.09–8.19)	0.04
PVD PRS^^^	NA	NA	0.9 (0.82–0.99)	0.02
Asthma	0.76 (0.57–1.02)	0.07	NA	NA
Cystatin C				
1	1	2.40 × 10^− 3^	1	6.43 × 10^− 4^
2	0.94 (0.61–1.46)		0.89 (0.56–1.42)	
3	1.18 (0.79–1.77)		1.23 (0.81–1.87)	
4	1.33 (0.89–1.97)		1.26 (0.84–1.91)	
5	1.48 (1–2.18)		1.53 (1.03–2.29)	
Diabetes	1.46 (1.13–1.89)	0.018562	1.53 (1.16–2.03)	0.007754
Alzheimers PRS^^^	1.14 (1.05–1.25)	2.54 × 10^− 3^	1.14 (1.04–1.25)	5.22 × 10^− 3^
Hypertension	1.35 (1.12–1.64)	7.81 × 10^− 3^	1.32 (1.08–1.6)	0.03
Comorbidity count*				
1	1	5.59 × 10^− 6^	1	2.51 × 10^− 4^
2	1.35 (1.07–1.69)		1.18 (2.10–1.49)	
3	1.80 (1.40–2.33)		1.59 (1.24–2.04)	

COVID-19 mortality: death within 100 days of COVID-19 diagnosis.

Clinico-demographic and PRS-adjusted model included age (as continuous), sex, ethnicity (in transethnic population), smoking status, Townsend deprivation quintile, immunosuppressant use, co-morbidities, anthropomorphic and biochemical traits (each as continuous), and PRS (as continuous). In this model, co-morbidity count is adjusted for these variables excepting the following comorbidities: diabetes and hypertension.

*Count of the following co-morbidities: cardiovascular disease, chronic respiratory disease, chronic kidney disease, diabetes, hypertension, chronic liver disease, neurological disease.

^^^Adjusted for top 10 principal components from principal component analysis.

*P*-value from the likelihood ratio test for association.

HR, odds ratio; CI, confidence intervals; *P*, *P*-value; LR, likelihood ratio; PRS, polygenic risk score; BMI, body mass index; BMR, basal metabolic rate; WHR, waist-hip ratio; AF, atrial fibrillation; PVD, peripheral vascular disease.

### Comparison of model fit

Model fit was compared between epidemiological models in this work, revealing that the addition of sociodemographic variables to the PRS model improved model fit (SeverityM1PRS AIC = 7361.77; SeverityM2SocioPRS AIC = 6332.91 [transethnic]), and the addition of clinical variables to SeverityM2SocioPRS further improved model fit (SeverityM2SocioPRS AIC = 6332.91; SeverityM4ClinicoDemPRS AIC = 6119.35 [transethnic]; Table 4).

**Table 4:** Akaike information criterion and Bayes information criterion values for each of the COVID-19 severity and COVID-19 mortality models, in both the transethnic and white European populations.

Model	COVID-19 severity						COVID-19 mortality					
	Transethnic			White European			Transethnic			White European		
	*N* variables in model	AIC	BIC	df	AIC	BIC	*N* variables in modela	AIC	BIC	df	AIC	BIC
PRS	8	7361.77	7415.95	8	6441.20	6494.29	2	8652.30	8660.75	3	7718.28	7730.65
Sociodemographic	11	6362.91	6437.42	9	5541.38	5601.11	10	8083.74	8125.97	8	7194.45	7227.45
Sociodemographic & PRS (M3SocioPRS)	15	6332.89	6434.49	13	5511.81	5598.08	13	8073.61	8128.50	12	7183.94	7233.43
Clinico-demographic (M4Clinical)	25	6111.58	6280.92	22	5327.84	5473.84	19	8029.34	8109.57	19	7148.80	7227.17
Clinico-demographic & PRS (M5ClinicoPRS)	21	6119.35	6261.59	23	5325.25	5477.89	19	8019.69	8099.92	18	7135.93	7210.17
Clinico-demographic & 10 PCs (M5ClinicoPRS with PRS substituted for PCs)	29	6127.74	6324.17	30	5336.03	5535.11	27	8041.36	8155.37	28	7137.44	7252.93

AIC, Akaike information criterion; BIC, Bayes information criterion; df, degrees of freedom; PRS, polygenic risk scores; PCs, principal components.

### Pathway analysis

Pathway analysis was performed (using FUMA v1.4.0) on PRS with severe COVID-19 or COVID-19 mortality associations in the SeverityM4ClinicoDemPRS or MortalityM4ClinicoDemPRS models (Supplementary Tables 22–24). This revealed several pathways of potential interest, including enrichment of SNPs in the 994,087 SNP hypertension PRS in the GO ‘voltage gated calcium channel activity involved in cardiac muscle cell action potential’ pathway (*N genes* = 5; *beta[SE]* = 3.48 [0.60]; *adjusted-P* = 1.86 × 10^− 5^). Other pathways highlighted were the KEGG ‘vascular smooth muscle contraction’ pathway (*N* genes in gene set = 115; *N* genes = 24; *adjusted-P* = 5.18 × 10^− 3^) and ‘gonadotropin-releasing hormone (GNRH) signalling’ pathway (*N* genes in gene set = 101; *N* genes present = 22; *adjusted-P* = 5.18 × 10^− 3^) in the Alzheimer’s disease PRS, and the GO ‘membrane repolarization’ pathway (*N genes* = 43; *beta[SE]* = 1.25[0.06]; *adjusted-P* = 4.45 × 10^− 13^), in the AF PRS. Further details may be found in Supplementary Results.

## Discussion

To our knowledge, this study is the first to successfully highlight associations between clinical trait PRS and poor COVID-19 outcomes even following adjustment for other sociodemographic and clinical variables, demonstrating the potential benefits of integrating genetic data into epidemiological models, alongside other risk factors. This work also shows the importance of investigating PRS of multiple clinical traits, which may exhibit stronger associations in models including sociodemographic and clinical variables, compared to using a single PRS optimized for the clinical outcome of interest. In addition to this, pathway analysis of the PRS retained in the fully-adjusted models revealed shared pathogenic mechanisms between several variables and COVID-19 disease, including ‘GNRH signaling’ and ‘cardiac muscle contraction’.

Univariate associations with COVID-19 severity and/or mortality were found for 17 trait PRS, and these PRS were included in a single model, and further adjusted for sociodemographic factors. The weak correlations found between regression coefficients of PRS in the model suggested that the retained PRS had limited overlap and independently contributed predictive value to the model not conferred by other PRS. Four of these associations remained following adjustment for both sociodemographic and clinical factors: the hypertension PRS, AF PRS, Alzheimer’s disease PRS and the PVD PRS. For three of these four results (hypertension, AF and Alzheimer’s disease), the association between the COVID-19 outcome and the PRS proxy of the trait (e.g. hypertension PRS) was stronger than that between the COVID-19 outcome and the trait itself (e.g. hypertension).

There are several reasons why some PRS might be more effective predictors of COVID-19 outcomes compared with their clinical counterparts in these models. Firstly, this enables the identification of individuals who may have a genetic predisposition to certain traits or diseases, even if they have not developed the disease or received a formal diagnosis. By incorporating this information, we can avoid overlooking individuals who may have been missed when relying solely on clinical data to establish associations. Furthermore, including this “at risk” information in the analysis in the form of a continuous predictor may improve the statistical power to detect associations, particularly when the clinical trait under consideration is traditionally defined as a binary variable. Secondly, inconsistencies between clinical definitions are evident in healthcare and epidemiology^7^. This can result in variation in disease definitions and therefore classifications of individuals in the study, particularly when collating information from self-reports or different healthcare settings. This may lead to inaccurate estimates of effect sizes when testing for associations with the clinical trait. Contrastingly, PRS are calculated systematically using a single algorithm, reducing the impact of bias or variation on classification of individuals and leading to greater consistencies when testing for associations within epidemiological studies. Thirdly, some variables, such as BMI and BF%, may be measured crudely in small epidemiological cohorts, whereas PRS for these traits may benefit from optimization using data from large, consistently measured datasets, improving their uniformity within the sample of interest. Nevertheless, it is important to acknowledge that clinical risk factors played a significant role in enhancing COVID-19 outcome models in this study. Therefore, it is advisable that PRS be considered as supplementary rather than substitutive components in such models when clinical variables are accessible.

The PVD PRS was present in an epidemiological model for COVID-19 mortality (MortalityM4ClinicoDemPRS) alongside its clinical counterpart, PVD. Both the PVD trait and the PVD PRS had a *LR P-value* < 0.05, suggesting that both traits independently contributed to risk of the COVID-19 mortality outcome in this study. These results provide further evidence that PRS may provide risk information above and beyond that of their clinical counterpart alone. However, it is noteworthy that the effect size of PVD and the PVD PRS were in opposing directions in this study. Several explanations may account for this outcome. Firstly, there may be unmeasured confounding influencing the effect of these traits on severe COVID-19. For example, pleiotropic SNPs in the PVD PRS could be influencing COVID-19 outcome risk through an alternate pathway to PVD itself. Likewise, PVD is a complex trait which is likely influenced by numerous genetic factors, each with differing effects on disease risk. The PVD PRS described here may capture just a subset of PVD risk, leading to discrepancies between the PRS effect size and the PVD effect size on severe COVID-19 risk. Finally, collider bias could be influencing this association due to the adjustment of genetic PCs. If genetic information in the PCs are also associated with other severe COVID-19 risk factors (e.g. blood group), the observed association between the PVD PRS and severe COVID-19 could be a type one error masking the true causal risk factor. Future studies may employ Mendelian randomization techniques to test for a causal relationship between PVD and COVID-19 outcomes through this PRS, as well as testing for potential confounding pathways.

As anticipated, the fit of the PRS model improved with the addition of sociodemographic and clinical variables. Interestingly, when comparing epidemiological models formed in this study, we observed that the fit of the sociodemographic & PRS model (SeverityM2SocioPRS) was better than a model containing sociodemographic variables alone. This was also found when comparing models with sociodemographic, clinical and PRS variables (SeverityM4ClinicoDemPRS) with just sociodemographic and clinical factors (SeverityM3ClinicoDem). Together, these results suggest that the addition of PRS could improve the fit of epidemiological models containing classic sociodemographic and/or clinical risk factors alone. Such findings should be further investigated in future epidemiological and risk prediction studies.

A statistically significant association was found between the 6,887 SNP Alzheimer’s disease PRS and COVID-19 mortality in the transethnic and white European MortalityM5ClinicoPRS models. This association had a positive direction of effect, wherein an increase in Alzheimer’s disease PRS was associated with an elevated risk of both Alzheimer’s disease and COVID-19 mortality, even after adjustment for other clinico-demographic variables. This PRS was enriched for SNPs in both the ‘GNRH signaling’ and ‘vascular smooth muscle cell contraction’ gene sets, highlighting a possible shared aetiology of Alzheimer’s disease and severe COVID-19 through the PRS’s effect on these biological pathways. These results highlight another potential benefit of testing for associations between trait PRS and disease outcomes in epidemiological modelling. By performing pathway analyses on genetic variants in the trait PRS, it is possible shed light on the pathogenic mechanisms underpinning predisposition to not only the trait itself, but also the disease outcome studied in the epidemiological model. However, results of such enrichment studies should be interpreted with caution, given that the inclusion of some false positive SNP associations are inherent to PRS methodologies^6^.

Shortcomings of this work included the limited availability of non-white European samples in the study cohort. Whilst the study attempted to repeat risk analyses in a transethnic population, because of the predominance of white European samples in the UK Biobank cohort^27^, this was difficult to conduct. PRS were therefore optimized in a white European population (to minimize issues related to population stratification), meaning that PRS may not be as effective at predicting risk of COVID-19 outcomes in non-European populations due to differences in LD structure and genetic architecture. This is representative of a wider problem in the genetics community, with work needed to recruit more diverse populations into cohort studies.

The optimization of PRS and prediction of risk in COVID-19 outcomes is also limited by statistical power in the current study, which is constrained by sample sizes of current datasets and the need for a complete case approach. For example, instances in this work wherein associations were found for risk factors in predicting COVID-19 severity but not COVID-19 mortality (e.g. hypertension PRS), could be in part due a loss of power in the smaller cohort sizes of the COVID-19 mortality outcome. Interestingly, the use of PRS as clinical proxies in future epidemiological studies could mitigate these issues, as this circumvents the issue of missing values for clinical variables.

It is also important to note that whilst associations were identified between PRS and COVID-19 outcomes after adjustment for clinico-demographic factors, the models created here are not risk prediction models. More work is needed before PRS are integrated in a clinical setting, including cross-validation studies^1,4,5,28^. This may be possible using other population-based cohorts such as 23andMe^29^ or the upcoming Our Future Health project in the UK^30^. Improvements in PRS performance will occur over time with increasing cohort sizes, particularly in transethnic populations.

This study identified associations between PRS for clinical traits (e.g. hypertension and AF) and poor COVID-19 outcomes, highlighting the value of including multiple trait PRS over a single PRS optimised for the outcome of interest, and identifying shared biological pathways between these traits. This work demonstrates that genetic data can improve the fit of sociodemographic models for COVID-19 outcomes, and highlights the potential benefits of incorporating PRS in disease modelling. As PRS for complex diseases are further refined, concurrent improvements in disease modelling will be attained.

## Electronic supplementary material

Below is the link to the supplementary material.

Supplementary Material 1

Supplementary Material 2

## Data Availability

UK Biobank data were provided under a licence that does not permit sharing data. The code-lists used in definitions and the derived results are published in Crossfield et al. (2022)^2^. UK Biobank data is available online (https://www.ukbiobank.ac.uk/). To request further information, please contact AW Morgan.

## References

[1] Slunecka, J. L. et al. Implementation and implications for polygenic risk scores in healthcare. *Hum. Genom.* **15** (1), 46 (2021). 10.1186/s40246-021-00339-y PMC8290135 34284826

[2] Crossfield, S. S. R., Chaddock, N. J. M., Iles, M. M., Pujades-Rodriguez, M. & Morgan, A. W. Interplay between demographic, clinical and polygenic risk factors for severe COVID-19. *Int. J. Epidemiol.* **51** (5), 1384–1395 (2022). 35770811 10.1093/ije/dyac137 PMC9278202

[3] Mars, N. et al. The role of polygenic risk and susceptibility genes in breast cancer over the course of life. *Nat. Commun.* **11** (1), 6383 (2020). 33318493 10.1038/s41467-020-19966-5 PMC7736877

[4] Weale, M. E. et al. Validation of an integrated risk tool, including polygenic risk score, for atherosclerotic cardiovascular disease in multiple ethnicities and ancestries. *Am. J. Cardiol.* **148**, 157–164 (2021). 33675770 10.1016/j.amjcard.2021.02.032

[5] England, H. E. NHS launches new polygenic scores trial for heart disease United Kingdom 2021.https://www.genomicseducation.hee.nhs.uk/blog/nhs-launches-new-polygenic-scores-trial-for-heart-disease/

[6] Choi, S. W., Mak, T. S. & O’Reilly, P. F. Tutorial: A guide to performing polygenic risk score analyses. *Nat. Protoc.* **15** (9), 2759–2772 (2020). 32709988 10.1038/s41596-020-0353-1 PMC7612115

[7] Hajjaj, F. M., Salek, M. S., Basra, M. K. & Finlay, A. Y. Non-clinical influences on clinical decision-making: A major challenge to evidence-based practice. *J. R Soc. Med.* **103** (5), 178–187 (2010). 20436026 10.1258/jrsm.2010.100104 PMC2862069

[8] Lee, C. M. Y. et al. Comparing different definitions of prediabetes with subsequent risk of diabetes: An individual participant data meta-analysis involving 76 513 individuals and 8208 cases of incident diabetes. *BMJ Open. Diabetes Res. Care* **7** (1), e000794 (2019). 31908797 10.1136/bmjdrc-2019-000794 PMC6936411

[9] Zhou, P. et al. A pneumonia outbreak associated with a new coronavirus of probable bat origin. *Nature* **579** (7798), 270–273 (2020). 32015507 10.1038/s41586-020-2012-7 PMC7095418

[10] Docherty, A. B. et al. Features of 20 133 UK patients in hospital with covid-19 using the ISARIC WHO clinical characterisation protocol: Prospective observational cohort study. *BMJ* **369**, m1985 (2020). 32444460 10.1136/bmj.m1985 PMC7243036

[11] Williamson, E. J. et al. Factors associated with COVID-19-related death using OpenSAFELY. *Nature* **584** (7821), 430–436 (2020). 32640463 10.1038/s41586-020-2521-4 PMC7611074

[12] McKeigue, P. M. et al. Rapid Epidemiological Analysis of Comorbidities and treatments as risk factors for COVID-19 in Scotland (REACT-SCOT): A population-based case-control study. *PLoS Med.* **17** (10), e1003374 (2020). 33079969 10.1371/journal.pmed.1003374 PMC7575101

[13] Initiative, C-H-G. Mapping the human genetic architecture of COVID-19. *Nature* **600** (7889), 472–477 (2021). 34237774 10.1038/s41586-021-03767-x PMC8674144

[14] Pairo-Castineira, E. et al. Genetic mechanisms of critical illness in COVID-19. *Nature* **591** (7848), 92–98 (2021). 33307546 10.1038/s41586-020-03065-y

[15] Ellinghaus, D. et al. Genomewide association study of severe Covid-19 with respiratory failure. *N. Engl. J. Med.* **383** (16), 1522–1534 (2020). 32558485 10.1056/NEJMoa2020283 PMC7315890

[16] Hu, J., Li, C., Wang, S., Li, T. & Zhang, H. Genetic variants are identified to increase risk of COVID-19 related mortality from UK Biobank data. medRxiv (2020). 10.1186/s40246-021-00306-7 PMC7856608 33536081

[17] Shelton, J. F. et al. Trans-ancestry analysis reveals genetic and nongenetic associations with COVID-19 susceptibility and severity. *Nat. Genet.* **53** (6), 801–808 (2021). 33888907 10.1038/s41588-021-00854-7

[18] Horowitz, J. E. et al. Genome-wide analysis provides genetic evidence that ACE2 influences COVID-19 risk and yields risk scores associated with severe disease. *Nat. Genet.* **54** (4), 382–392 (2022). 35241825 10.1038/s41588-021-01006-7 PMC9005345

[19] Dite, G. S., Murphy, N. M. & Allman, R. An integrated clinical and genetic model for predicting risk of severe COVID-19: A population-based case-control study. *PLoS One* **16** (2), e0247205 (2021). 33592063 10.1371/journal.pone.0247205 PMC7886160

[20] Brown, K. L., Ramlall, V., Zietz, M., Gisladottir, U. & Tatonetti, N. P. Estimating the heritability of SARS-CoV-2 susceptibility and COVID-19 severity. *Nat. Commun.* **15** (1), 367 (2024). 38191623 10.1038/s41467-023-44250-7 PMC10774300

[21] Sudlow, C. et al. UK biobank: An open access resource for identifying the causes of a wide range of complex diseases of middle and old age. *PLoS Med.* **12** (3), e1001779 (2015). 25826379 10.1371/journal.pmed.1001779 PMC4380465

[22] Team, R. C. R: A language and environment for statistical computing Vienna, Austria: R Foundation for Statistical Computing. https://www.R-project.org/ (2019).

[23] Chang, C. C. et al. Second-generation PLINK: Rising to the challenge of larger and richer datasets. *Gigascience* **4**, 7 (2015). 25722852 10.1186/s13742-015-0047-8 PMC4342193

[24] Choi, S. W. & O’Reilly, P. F. PRSice-2: Polygenic risk score software for biobank-scale data. *Gigascience* **8** (7) (2019). 10.1093/gigascience/giz082 PMC6629542 31307061

[25] Watanabe, K., Taskesen, E., van Bochoven, A. & Posthuma, D. Functional mapping and annotation of genetic associations with FUMA. *Nat. Commun.* **8** (1), 1826 (2017). 29184056 10.1038/s41467-017-01261-5 PMC5705698

[26] de Leeuw, C. A., Mooij, J. M., Heskes, T. & Posthuma, D. MAGMA: Generalized gene-set analysis of GWAS data. *PLoS Comput. Biol.* **11** (4), e1004219 (2015). 25885710 10.1371/journal.pcbi.1004219 PMC4401657

[27] Bycroft, C. et al. The UK Biobank resource with deep phenotyping and genomic data. *Nature* **562** (7726), 203–209 (2018). 30305743 10.1038/s41586-018-0579-z PMC6786975

[28] Sollis, E. et al. The NHGRI-EBI GWAS catalog: Knowledgebase and deposition resource. *Nucleic Acids Res.* **51** (D1), D977–D85 (2023). 36350656 10.1093/nar/gkac1010 PMC9825413

[29] 23andMe. 23andMe 2022. https://www.23andme.com/en-gb/

[30] Health NIo. All of Us Research Program (2023).

