# Supplementary material for: Genetic proxies for clinical traits are associated with increased risk of severe COVID-19
Source: Sci Rep. 2025 Jan 15;15:2083. doi: 10.1038/s41598-025-86260-z (PMC11736077; doi:10.1038/s41598-025-86260-z)
Supplement: Supplementary file 1 — Supplementary Material 1 [file 41598_2025_86260_MOESM1_ESM.docx]

**Supplementary Methods**

Trait Selection

The literature search engines PUBMED, MEDLINE and Web of Science were used to search for traits related to risk factors included in the Crossfield *et al.* (2022) [1] models, on which we based the variables selected for study in this work. Of these traits, those that were available in UK Biobank were included in downstream analyses and, where possible, polygenic risk scores (PRS) were created (**Supplementary Table 1**).

Investigated risk factors included those defined previously [1]: socio-demographic factors including age, sex, smoking status, Townsend deprivation index [2] and ethnicity; immunosuppressant use; body mass index (BMI); comorbidity count and individual comorbidities including cardiovascular disease (CVD), chronic respiratory disease (CRD), chronic kidney disease (CKD), chronic liver disease (CLD), diabetes, hypertension, neurological disease and autoimmune disease; and a previously developed COVID-19 PRS optimized in a white European population (hereafter named “PRS_e2_”, maintaining the nomenclature used in our original publication) [1].

Additional risk factors that were studied encompassed traits defined by baseline diagnoses in UK Biobank. These included BMI-related traits such as basal metabolic rate (BMR) (in kj), waist-hip ratio (WHR), weight (in kg) and body fat percentage (BF%); the age-related leukocyte telomere length trait [3], albumin (g/L) (a biomarker of liver synthetic function), cystatin C (mg/L) as an alternative biomarker of renal function and individual CRD disorders including emphysema, asthma and chronic obstructive pulmonary disease (COPD).

Diabetes subtypes one (T1D) and two (T2D) were investigated (further details of diabetes subtype definitions are provided below), as was the related trait glycated haemoglobin (HbA1c) (mmol/mol). Note that the HbA1c GWAS and PRS were calculated in all individuals without a diagnosis of diabetes nor coded medications for diabetes in UK Biobank data. Other studied traits included Alzheimer’s disease as a subtype of neurological disease and specific CVD traits, such as transient ischaemic attack (TIA), thromboembolic disease, atrial fibrillation (AF), peripheral vascular disease (PVD), heart failure (HF), myocardial infarction (MI), stroke and angina.

Diabetes definition

Because the numbers of those with self-reported T1D and T2D in the UK Biobank baseline data were smaller than expected (*N*=438 and *N*=3,375, respectively), and together comprised only 13.87% of those cases who reported “diabetes” (3,813/27,496; UK Biobank field 2976), a novel definition of T1D and T2D was devised using additional information from UK Biobank baseline data.

According to previous studies, most individuals with T1D are prescribed insulin, whereas those with T2D are more commonly prescribed metformin and other oral hypoglycemics [4, 5]. Likewise, although a small proportion of T1D cases are diagnosed at mid-life, most are thought to be identified during adolescence [6]. T2D is most often diagnosed later in life, akin to other lifestyle-related diseases [7]. Therefore, a novel index for diabetes subtypes was constructed using the baseline data fields: “started insulin within one year diagnosis of diabetes” (2986) and “age diabetes diagnosed” (2976), along with the “diabetes” response in field 20002. The “age diabetes diagnosed” distribution was first observed for known (self-reported in baseline field 20002) T1D and T2D samples, along with binary response to variable “started insulin within one year diagnosis of diabetes”. It was found that the definition that captured a majority (56.39%) of the total self-reported T1D samples was “age of onset under 50 and started insulin within 1 year of diagnosis” (*N*=247), whilst the definition that captured a majority (75.76%) of the total self-reported T2D samples was “age of onset over 40 and did not start insulin within 1 year of diagnosis” (*N*=2,557) (**Supplementary Table 2**). Finally, these definitions were applied to all samples that reported “diabetes” in field 20002 of UK Biobank baseline data, resulting in 1,700 total T1D samples and 12,453 total T2D samples.

Construction of polygenic risk scores

Polygenic risk scores (PRS) were optimized for clinical traits investigated in this work. Where genome-wide association study (GWAS) results were available in public databases, PRS were constructed using effect sizes and *P-values* from the published summary statistics. It is important to note that some samples used in these GWAS could overlap with the severe COVID-19 testing cohort, potentially resulting in estimates of the test statistic being upwardly biased. Therefore, where traits with potential overlaps produced PRS that were associated with severe COVID-19, the PRS was retested for association with severe COVID-19 using summary statistics from a GWAS performed in UK Biobank (**Supplementary Table 1**). These GWAS were performed using an equivalent disease definition in UK Biobank, with samples from the COVID-19 dataset removed.

For traits where summary statistics weren’t publicly available, a definition for said trait was formed using data from UK Biobank, and a GWAS was performed using this phenotype. In these instances, samples from the COVID-19 dataset were removed prior to GWAS and PRS analyses to ensure independence.

UK Biobank Genetic Quality Control

Details of the quality control (QC) of UK Biobank genetic data used in this work have previously been described in Crossfield *et al.* (2022) [1]. Genotyping and imputation were performed centrally by UK Biobank [8], using either the Affymetrix UK BiLEVE Axiom or the Affymetrix UK Biobank Axiom array for each individual. Combined reference panels from the Haplotype Reference Consortium [9], 1000 Genomes [10] and UK10K [11] projects were used to impute ~90 million variants. Sample and variant-based quality control thresholds were applied to genetic data, as outlined previously [1], and the definition of the white European sub-cohort using principal component analysis (PCA) was applied, as previously described.

Genome-Wide Association Studies

A GWAS was performed for each risk factor using data from UK Biobank (**Supplementary Table 1**). An important assumption of conventional PRS construction is that no sample overlap is present between the training (risk factor GWAS) and testing (COVID-19 outcome) cohorts [12]. Such sample overlap could result in inflated test statistics [13]. GWAS was conducted for 27 phenotypes in a white European UK Biobank sample, using either logistic or linear regression in PLINK v1.9 [14]. Any samples present in the COVID-19 case-control cohort were excluded from these analyses, and the top 10 PCs from PCA were included as covariates. Finally, significant SNPs were defined as those that surpassed the genome-wide threshold for statistical significance (*P*<5 x 10^-8^) with GWAS independent signals declared for SNPs with a linkage disequilibrium (LD) *R^2^*<0.6 (for informative purposes only).

Polygenic Risk Score Analyses

PRSice v2.0 (16) was used to construct PRS using effect sizes and *P-values* from GWAS performed for clinical traits. No pre-defined filter was applied to these GWAS summary statistics prior to clumping. The “clumping and thresholding” approach was used to optimise PRS, with the top 10 PCs from the PCA included as covariates. In the clumping stage, loci were thinned in 250kb blocks, prioritizing variants with the smallest GWAS *P-values*, and based on a LD *R^2^* threshold of 0.1. All SNPs with a *P-value* below a specified threshold (*P_T_*) in the summary statistics were used to form a risk score, on which the severe COVID-19 case-control variable (“severe COVID-19” cases *N*=1,908, “non-severe COVID-19” controls *N*=5,366) was then regressed using an independent cohort. This process was repeated for multiple values *P_T_* (minimum *P_T_*=5 x 10^-8^; step size=5 x 10^-5^; maximum *P_T_*=1), producing a large number of risk scores from which to select the optimum. The “best fit” risk score was defined as the PRS with the strongest *P-value* association with severe COVID-19 in a logistic regression. Finally, an empirical *P-value* was produced using a permutation test, in order to account for the testing of multiple (correlated) risk scores for each phenotype.

Statistical modelling

Logistic regression was performed to model the risk of severe COVID-19, and Cox proportional hazards regression was used to model the risk of death within 100 days of diagnosis. In Cox regression, age was modelled as cubic splines (3 knots), with hazard ratios reported for ages 55, 60, 65, 70 and 75 years. The proportional hazards assumption was assessed for each variable in Cox regression using the Schoenfeld residuals. To reduce the number of degrees of freedom in our models, all non-binary risk factors were modelled as continuous variables in this study.

Univariate regression of polygenic risk scores and adjustment for sociodemographic factors

PRS were tested for association with each COVID-19 outcome in univariate analyses and PRS with a likelihood ratio (LR) test *P-value* of <0.05 were combined into separate PRS models of severe COVID-19 (SeverityM1PRS), or COVID-19 mortality (MortalityM1PRS). Correlations were assessed between regression coefficients of traits in these models, and one trait from each pairwise correlation with an *R^2^* > 0.8 was removed from the model, informed by a review of the literature to retain the most clinically and epidemiologically relevant trait.

Backwards stepwise regression was then performed on the SeverityM1PRS and MortalityM1PRS models to remove redundant variables, using the Akaike Information Criterion (AIC) of the MASS R package. Finally, PRS were adjusted for sociodemographic variables, creating the SeverityM2SocioPRS and MortalityM2SocioPRS models.

Adjustment of polygenic risk score associations for clinical factors

In order to select the relevant clinical variables to adjust PRS associations for, clinical variables were first tested for association with COVID-19 outcomes in univariate analyses. PRS with a likelihood ratio (LR) test *P-value* of < 0.05 were added to a model including other clinical variables and socio-demographic risk factors. A correlation matrix was produced using the regression coefficients from this model, and one variable from each pair with a regression coefficient correlation *R^2^* ≥ 0.8 was removed. Backwards stepwise regression was performed on the models using StepAIC in the MASS R package, creating the SeverityM3ClinicoDem and MortalityM3ClinicoDem.

Pathway Analysis

PRS with COVID-19 severity or mortality associations in SeverityM4ClinicoDemPRS or MortalityM4ClinicoDemPRS were used as input for pathway analysis. The Functional Mapping and Annotation of Genome-Wide Association Studies (FUMA) v1.4.0 tool [15] was used to perform pathway analyses, which combines information from multiple *in silico* tools to provide biological interpretation of SNPs in the PRS. These tools include the Multi-marker Analysis of GenoMic Annotation (MAGMA) gene-based test [16], which assesses the joint effects of genetic markers in a gene to test for an association between the gene and phenotype. The MAGMA gene-set analysis then uses these gene-level associations to perform gene-set enrichment analysis (GSEA), revealing more information about the functional mechanisms underlying the associations. GSEA was also performed using data from the Reactome [17] database, to provide additional biological insight.

**Supplementary Results**

**Genome-Wide Association Study Results**

For several UK Biobank traits, genome-wide association studies (GWAS) were performed to produce effect sizes for the construction of PRS. A total of 42,353 SNP associations passed the *P-value*<5 x 10^-8^ statistical significance threshold across 29 traits (**Supplementary Table 1**).

**Univariate polygenic risk score associations and adjustment for sociodemographic variables**

COVID-19 Severity

Associations were identified between 14 PRS and severe COVID-19 in univariate regression (**Supplementary Tables 3-16**), including the BMI PRS (OR=1.46, 95%CI 1.23-1.58, *P* <0.001 [transethnic], OR=1.48, 95%CI 1.23-1.79, *P* <0.001 [white European]), hypertension PRS (OR=1.57, 95%CI 1.31-1.87, *P* <0.001 [transethnic], OR=1.5, 95%CI 1.24-1.81, *P* <0.001 [white European]) and stroke PRS (OR=1.32, 95%CI 1.11-1.57, *P* <0.001 [transethnic], OR=1.37, 95%CI 1.14-1.66, *P* <0.001 [white European]).

PRS with a LR P<0.05 in univariate analyses were combined into the SeverityM1PRS model (**Table 1**). To measure the extent of colinearity amongst PRS in this model, correlations between regression coefficients of PRS in the model were assessed. No regression coefficients had a pairwise correlation *R^2^*>0.8, meaning that no PRS was removed from the model at this stage.

Following backwards stepwise regression, seven PRS remained in both the transethnic and white European SeverityM1PRS (**Supplementary Table 17**). Of these PRS, three were associated with severe COVID-19 risk in the transethnic population, even after adjustment for other PRS and non-genetic factors. This included the BMI PRS (AOR=1.1, 95%CI 1.03-1.17, *P*=2.17 x 10^-3^ [transethnic]; AOR=1.11, 95%CI 1.04-1.18, *P*=1.33 x 10^-3^ [white European] with increasing PRS), the stroke PRS (AOR=1.08, 95%CI 1.02-1.14, *P*=7.80x 10^-3^ [transethnic]; AOR=1.1, 95%CI 1.04-1.17, *P*=1.54 x 10^-3^ [white European] with increasing PRS) and the hypertension PRS (AOR=1.1, 95%CI 1.04-1.16, *P*=1.71 x 10^-3^ [transethnic]; AOR=1.09, 95%CI 1.02-1.16, *P*=0.01 [white European] with increasing PRS).

PRS with a LR *P*<0.05 in this model were further adjusted for socio-demographic factors in SeverityM2SocioPRS (**Supplementary Table 17**), and all three PRS remained associated with severe COVID-19 in the transethnic and/or white European population: the BMI PRS (adjusted odds ratio [AOR]=1.14 95% confidence intervals [CI] 1.07-1.21, *P-value* [*P*] =9.51 x 10^-5^ [transethnic]; AOR=1.15, 95%CI 1.07-1.23, *P*=8.00 x 10^-5^ [white European]), hypertension PRS (AOR=1.11, 95%CI 1.04-1.18, *P*=2.63 x 10^-3^ [transethnic]; AOR=1.09, 95%CI 1.02-1.17, *P*=0.01 [white European]), and the stroke PRS (AOR=1.08, 95%CI 1.01-1.15, *P*=0.02 [white European]).

COVID-19 Mortality

Associations were identified between three PRS and COVID-19 mortality in univariate regression (**Supplementary Tables 3-16**), including the AF PRS (OR=1.15, 95%CI 1.06-1.26, *P*=0.001 [transethnic], OR=1.15, 95%CI 1.05-1.26, *P*=0.002 [white European]), Alzheimer’s disease PRS (OR=1.16, 95%CI 1.07-1.26, *P*<0.001 [transethnic], OR=1.16, 95%CI 1.06-1.26, *P*=0.002 [white European]) and PVD PRS (OR=0.9, 95%CI 0.83-0.99, *P*=0.04 [white European]).

Correlations between regression coefficients of PRS in the MortalityM1PRS model were assessed. No regression coefficients had a pairwise correlation R^2^>0.8, and no PRS were removed from the model at this stage.

Three PRS were retained in the MortalityM1PRS model (AF PRS, PVD PRS and Alzheimer’s PRS), and each had an association (LR *P*<0.05) with COVID-19 mortality in the transethnic and/or white European population (**Supplementary Table 18**). All three PRS remained associated with COVID-19 mortality following adjustment for sociodemographic factors in MortalityM2SocioPRS: AF PRS (AOR=1.12, 95%CI 1.03-1.22, *P*=0.01 [transethnic]; AOR=1.11, 95%CI 1.02-1.22, *P*=0.02 [white European]), (AOR=1.14, 95%CI 1.05-1.24, *P*=2.50 x 10^-3^ [transethnic]; AOR=1.14, 95%CI 1.04-1.25, *P*=4.44 x 10^-3^ [white European]), and PVD PRS (AOR=0.9, 95%CI 0.83-0.99, *P*=0.03 [white European]).

**Adjustment of polygenic risk score associations for clinical variables**

COVID-19 Severity

In order to select the relevant clinical risk factors for which to adjust PRS associations, univariate regression was first performed on clinical variables, identifying 29 associations with severe COVID-19, including: leukocyte telomere length (OR=0.44, 95%CI 0.36-0.52, *P*<0.001 [transethnic], OR=0.43, 95%CI 0.36-0.52, *P*<0.001 [white European]), cystatin C (OR=7.26, 95%CI 5.92-8.91, *P*<0.001 [transethnic], OR=7.11, 95%CI 5.72-8.84, *P*<0.001 [white European]), and HbA1c (OR=4.18, 95%CI 3.46-5.05, *P*<0.001 [transethnic], OR=3.97, 95%CI 3.25-4.84, *P*<0.001 [white European]).

To determine whether there was likely to be collinearity between clinical traits (**Supplementary Table 19**) in the SeverityM3ClinicoDem model, pairwise Pearson correlations were calculated between regression coefficients of the traits. Correlations with an *R^2^*>0.8 were found between the regression coefficients of BF% and BMR (*R^2^*=0.85 [transethnic]), BF% and weight (*R^2^*=-0.82 [transethnic]), BMR and weight (*R^2^*=-0.95 [transethnic]), and T2D and diabetes (*R^2^*=-0.81 [white European]). Of these variables, BMR and weight were removed, given that previous work has suggested that BF% is a more effective clinical indicator of adiposity and health than i) weight, which is confounded as a measure of adiposity or health by the varying presence of muscle and bone tissue, and excess water; and ii) BMR, which, although proportional to measures of fat-free mass, can be confounded by factors including acute illness and hormones related to menstruation in females [18, 19]. T2D was also removed from further analyses due to having: i) a smaller number of cases than diabetes, and ii) a weaker association with severe COVID-19.

Following backwards stepwise regression, several risk factors were associated with severe COVID-19 in the transethnic and/or white European cohort, even after adjustment for sociodemographic traits (**Supplementary Table 20**). These included the diabetes-related trait HbA1c (AOR=1.46, 95%CI 1.16-1.84, *P*=1.87 x 10^-4^ [transethnic] and AOR=1.34, 95%CI 1.05-1.71, *P*=7.15 x 10^-3^ [white European] with increasing blood HbA1c levels), leukocyte telomere length (AOR=0.82, 95%CI 0.67-1.01, *P*=4.55 x 10^-3^ [transethnic] and AOR=0.83, 95%CI 0.67-1.04, *P*=0.01 [white European]) and the biomarker cystatin C (AOR=2.14, 95%CI 1.69-2.73, *P*=2.05 x 10^-9^ [transethnic] and AOR=2.02, 95%CI 1.56-2.61, *P*=6.56 x 10^-9^ [white European]). Weight-related traits were associated with severe COVID-19, including BF% (AOR=1.64, 95%CI 1.19-2.27, *P*=1.04 x 10^-3^ [white European]), as were CRD subtypes, including emphysema (AOR=1.91, 95%CI 1.21-3.04, *P*=4.57 x 10^-3^ [transethnic] and AOR=1.87, 95%CI 1.16-3.05, *P*=0.01 [white European]).

COVID-19 Mortality

Univariate regression was also performed on clinical variables for the COVID-19 mortality phenotype, identifying 21 associations, including: leukocyte telomere length (OR=0.32, 95%CI 0.24-0.43, *P*<0.001 [transethnic], OR=0.32, 95%CI 0.23-0.43, *P*<0.001 [white European]), cystatin C (OR=6.25, 95%CI 4.37-8.94, *P*<0.001 [transethnic], OR=6.07, 95%CI 4.2-8.78, *P*<0.001 [white European]), and HbA1c (OR=3.78, 95%CI 2.77-5.17, *P*<0.001 [transethnic], OR=3.8, 95%CI 2.73-5.28, *P*<0.001 [white European]).

Correlations with an *R^2^*>0.8 were found between the regression coefficients of BMR and sex (*R^2^*=-0.82 [transethnic]). BMR was therefore removed from further analyses, since sex has been repeatedly established as an important risk factor for poor COVID-19 outcomes [1]. Numerous risk factors were associated with COVID-19 mortality in the transethnic and/or white European cohort following backwards stepwise regression, even following adjustment for other clinico-demographic traits (**Supplementary Table 21**). These included leukocyte telomere length (AOR=0.67, 95%CI 0.5-0.91, *P*=1.16 x 10^-4^ [transethnic] and AOR=0.68, 95%CI 0.49-0.94, *P*=4.82 x 10^-4^ [white European]), cystatin C (AOR=1.42, 95%CI 0.96-2.1, *P*=7.54 x 10^-3^ [transethnic] and AOR=1.41, 95%CI 0.94-2.10, *P*=2.89 x 10^-3^ [white European]), PVD (AOR=2.77, 95%CI 1.02-7.51, *P*=0.04 [transethnic] and AOR=2.89, 95%CI 1.06-7.88, *P*=0.04 [white European]) and WHR (AOR=1.34, 95%CI 0.85-2.12, *P*=0.02 [white European]).

**Clinical and sociodemographic-adjusted polygenic risk score associations with COVID-19 severity**

Several risk factors were associated with COVID-19 severity in the transethnic and/or white European SeverityM4ClinicoDemPRS (**Table 2**). These included the hypertension PRS (AOR=1.1, 95%CI 1.03-1.18, *P*=4.83 x 10^-3^ [transethnic]), baseline HbA1c (AOR=1.73, 95%CI 1.4-2.16, *P*=4.41 x 10^-10^ [transethnic]; AOR=1.34, 95%CI 1.06-1.71, *P*=7.31 x 10^-3^ [white European]), leukocyte telomere length (AOR=0.82, 95%CI 0.67-1, *P*=3.28 x 10^-3^ [transethnic] AOR=0.84, 95%CI 0.67-1.04, *P*=0.02 [white European]) and cystatin C (AOR=2.19, 95%CI 1.73-2.79, *P*=5.30 x 10^-11^ [transethnic] and AOR=2.00, 95%CI 1.55-2.59, *P*=9.05 x 10^-09^ [white European]). Additionally, the CRD subtypes emphysema (AOR=2.03, 95%CI 1.31-3.27, *P*=1.71 x 10^-3^ [transethnic] and AOR=1.85, 95%CI 1.15-3.02, *P*=0.01 [white European]) and COPD (AOR=4.22, 95%CI 1.84-11, *P*=7.01 x 10^-4^ [transethnic] and AOR=4.38, 95%CI 1.83-12.23, *P*=1.10 x 10^-3^ [white European]) were associated with risk of severe COVID-19. Finally, the body composition variables BF% (AOR=1.56, 95%CI 1.12-2.16, *P*=4.44 x 10^-3^) and WHR (AOR=1.42, 95%CI 1.04-1.95, *P*=0.04) were associated with risk of severe COVID-19 in the white European population.

**Clinical and sociodemographic-adjusted polygenic risk score associations with COVID-19 mortality**

In the MortalityM4ClinicoDemPRS model, several risk factors were associated with COVID-19 mortality in the transethnic and/or white European cohort (**Table 3**). These included the AF PRS (AOR=1.12, 95%CI 1.03-1.22, *P*=9.98 x 10^-3^ [transethnic] AOR=1.13, 95%CI 1.03-1.23, *P*=0.11 [white European]) the Alzheimer's PRS (AOR=1.14, 95%CI 1.05-1.25, *P*=2.54 x 10^-3^ [transethnic] and AOR=1.14, 95%CI 1.04-1.25, *P*=5.22 x 10^-3^ [white European]), and the PVD PRS (AOR=0.9, 95%CI 0.82-0.99, *P*=0.02 [white European]). The PVD trait itself was also associated with COVID-19 mortality (AOR=3.28, 95%CI 1.21-8.89, *P*=0.03 [transethnic] and AOR=2.99, 95%CI 1.09-8.19, *P*=0.04 [white European]), as was leukocyte telomere length (AOR=0.68, 95%CI 0.5-0.92, *P*=1.42 x 10^-4^ [transethnic] and AOR=0.68, 95%CI 0.49-0.94, *P*=4.56 x 10^-4^ [white European]), cystatin C (AOR=1.48, 95%CI 1-2.18, *P*=2.40 x 10^-3^ [transethnic] and AOR=1.53, 95%CI 1.03-2.29, *P*=7.75 x 10^-3^ [white European]), and WHR (AOR=1.36, 95%CI 0.86-2.15, *P*=0.02 [white European]).

**Pathway Analyses**

Hypertension PRS

Pathway analysis was performed using the 994,087 SNPs present in the hypertension PRS. Gene set enrichment analysis of the hypertension PRS using the MAGMA gene-set test revealed 10 pathways that were significantly enriched for SNPs in the risk score (**Supplementary Table 22**), including the GO ‘voltage gated calcium channel activity involved in cardiac muscle cell action potential’ pathway (*N* genes=5; *beta[SE]*= 3.48 [0.60]; *adjusted-P*=1.86 x 10^-5^) and the GO ‘ribbon synapse’ pathway (*N* genes=9; *beta[SE]*= 2.01 [0.40]; *adjusted-P*=3.91 x 10^-3^). Alzheimer's PRS

Using the FUMA v1.4.0 software [15], pathway analysis was performed on the 6,887 SNP Alzheimer’s disease risk score. Three gene sets were enriched for genes in the Alzheimer’s PRS, according to the Kyoto Encyclopedia of Genes and Genomes (KEGG) database (**Supplementary Table 23**). These gene sets included the ‘vascular smooth muscle contraction’ pathway (*N* genes in gene set=115; *N* genes present=24; *adjusted-P*=5.18 x 10^-3^) and the ‘GNRH signaling’ pathway (*N* genes in gene set=101; *N* genes present=22; *adjusted-P*=5.18 x 10^-3^).

AF PRS

The 34 SNP AF PRS was input to pathway analysis using FUMA v1.4.0 [15]. In the MAGMA gene-based test, 190 genes were highlighted as significantly enriched for SNPs in the AF PRS, at the Bonferroni-corrected statistical significance threshold (*P*=0.05/20,000=2.500 x 10^-6^). These genes included *SH3PXD2A* (*adjusted-P*=1.74 x 10^-26^), *KCNN3* (*adjusted-P*=1.49 x 10^-22^) and *PHLDB2* (*adjusted-P*=4.14 x 10^-21^) genes. Based on these results, 123 pathways were significantly enriched in the MAGMA gene-set analysis (**Supplementary Table 24**). This included the GO ‘cardiac muscle contraction’ pathway (*N genes*=132; *beta[SE]*=0.74[0.09]; *adjusted-P*=4.63 x 10^-14^) and the GO ‘membrane repolarization’ pathway (*N genes*=43; *beta[SE]*=1.25[0.06]; *adjusted-P*=4.45 x 10^-13^).

PVD PRS

Pathway analysis was performed on the 24,564 SNP PVD PRS using *in silico* tools collated in the FUMA software. Gene-set enrichment analysis identified just one Reactome pathway which had a statistically significant association with PVD (after multiple testing correction) via genes associated with PVD in the gene-based test. This was the ‘Transmission across chemical synapses’ pathway (*Gene set N*=264; *Genes in PRS*=25; *adjusted-P*=8.98 x 10^-3^).

References

1. Crossfield SSR, Chaddock NJM, Iles MM, Pujades-Rodriguez M, Morgan AW. Interplay between demographic, clinical and polygenic risk factors for severe covid-19. *Int J Epidemiol*. 2022;**51**(5):1384-95.

2. Townsend P, Phillimore P, Beattie A. *Inequalities in health in the northern region : An interim report*: NRHA; 1986.

3. Codd V, Denniff M, Swinfield C. Measurement and initial characterization of leukocyte telomere length in 474,074 participants in uk biobank. *Nat Aging*  2022. p. 170–9.

4. Excellence NIfHaC. Type 1 diabetes in adults: Diagnosis and management. 2015.

5. Excellence NIfHaC. Type 2 diabetes in adults: Management 2015 [Available from: <https://www.nice.org.uk/guidance/ng28>.

6. DiMeglio LA, Evans-Molina C, Oram RA. Type 1 diabetes. *Lancet*. 2018;**391**(10138):2449-62.

7. Sattar N, et al. Age at diagnosis of type 2 diabetes mellitus and associations with cardiovascular and mortality risks. *Circulation*. 2019;**139**(19):2228-37.

8. Bycroft C, et al. The uk biobank resource with deep phenotyping and genomic data. *Nature*. 2018;**562**(7726):203-9.

9. McCarthy S, et al. A reference panel of 64,976 haplotypes for genotype imputation. *Nat Genet*. 2016;**48**(10):1279-83.

10. Auton A, et al. A global reference for human genetic variation. *Nature*. 2015;**526**(7571):68-74.

11. Walter K, et al. The uk10k project identifies rare variants in health and disease. *Nature*. 2015;**526**(7571):82-90.

12. Choi SW, Mak TS, O'Reilly PF. Tutorial: A guide to performing polygenic risk score analyses. *Nat Protoc*. 2020;**15**(9):2759-72.

13. Wray NR, et al. Pitfalls of predicting complex traits from snps. *Nat Rev Genet*. 2013;**14**(7):507-15.

14. Chang CC, et al. Second-generation plink: Rising to the challenge of larger and richer datasets. *Gigascience*. 2015;**4**:7.

15. Watanabe K, Taskesen E, van Bochoven A, Posthuma D. Functional mapping and annotation of genetic associations with fuma. *Nat Commun*. 2017;**8**(1):1826.

16. de Leeuw CA, Mooij JM, Heskes T, Posthuma D. Magma: Generalized gene-set analysis of gwas data. *PLoS Comput Biol*. 2015;**11**(4):e1004219.

17. Jassal B, et al. The reactome pathway knowledgebase. *Nucleic Acids Res*. 2020;**48**(D1):D498-D503.

18. Curtis V, Henry CJK, Birch E, Ghusain-Choueiri A. Intraindividual variation in the basal metabolic rate of women: Effect of the menstrual cycle. *Am J Hum Biol*. 1996;**8**(5):631-9.

19. Kellerman R, Bope E. Conn's current therapy 2018, 1e. Elsevier; 2018.
